# Predictive hematological and immunological parameters associated with postpartum progressed Covid-19 disease

**DOI:** 10.4314/ahs.v21i4.10

**Published:** 2021-12

**Authors:** Fatma Bozkurt, Omer Coskun, Sevda Yelec

**Affiliations:** 1 Department of Infectious Diseases and Clinical Microbiology, University of Health Science, Gazi Yaşargil Education and Research Hospital, Diyarbakir, Turkey; 2 Department of Obstetrics and Gynecology, University of Health Science, Gazi Yaşargil Education and Research Hospital, Diyarbakir, Turkey

**Keywords:** COVID-19, delivery, albumin, neutrophil-lymphocyte ratio, pregnancy

## Abstract

**Background:**

In pregnancy, Coronavirus disease-2019 (COVID-19) infection disease may be more severe due to existing physiological changes. Similarly, changes during and after birth can make the patient more subceptible.

**Objective:**

To investigate possible laboratory findings that was related to postpartum progression of COVID-19 disease.

**Methods:**

Pregnant women who are pregnant at 28 weeks or more and who are COVID-19 positive at the time of delivery were investigated in this study. Progressed post- delivery and non-progressed COVID-19 positive pregnants' laboratory findings were analyzed. Hematological and immunological parameters associated with postpartum progressed COVID-19 disease were evaluated.

**Results:**

Totally 151 individuals were conducted to the study. In the prenatal analysis, higher BMI and lower albumin levels were detected in the progressed group (p<0.05). In the postpartum analysis; White Blood Cell, lymphocyte and albumin were increased, while neutrophil, NLR, LDH, CK, D-DIMER, Ferritin, CRP and IL-6 were decreased in the non-progressed group as opposite of the progressed group (p<005).

**Conclusion:**

We observed that prenatal low albumin and high BMI may be related to progression of the COVID-19 disease after delivery. In progressed group, inflammatory markers were increased after delivery while in non-progressed group they were improved. These markers may be warning for the postpartum progression of COVID-19 disease.

## Introduction

Coronavirus disease 2019 (COVID-19), which is caused by severe acute respiratory syndrome coronavirus 2 (SARS-CoV-2), is highly infectious, and every day, new information is learned about this virus and the disease it causes. As a result of the progression of the virus in different individuals with a different prognosis, clinical manifestations may occur that are difficult to predict. Cardiovascular and immune system adaptation in pregnancy make women more vulnerable to infections.^1^ Respiratory adaptations of pregnancy, such as diaphragmatic elevation, oedema of the respiratory tract mucosa and increased oxygen consumption, may increase the incidence of respiratory diseases, such pneumonia.^1^ This situation causes clinicians to be more concerned about the effect of COVID-19 on pregnant women. Furthermore, it is still unclear how this disease will progress in relation to the physiological changes that occur after giving birth.

Several studies have reported on the postpartum progression of COVID-19. One study found that 6 cases that were mild and 1 case that was severe at admission all aggravated one week after delivery,^2^ and different case reports found postpartum exacerbation.^3^,^4^ A report from Iran mentioned deterioration of COVID-19 after delivery and reported that 7 women died due to multiorgan deficiency and cardiopulmonary arrest.^5^ In a study published in Brazil, where the death rates due to COVID-19 are high, 50 (40. 3%) of 124 maternal deaths were seen in women with postpartum disease progression.^6^ The pathophysiology of postpartum progression is unclear. Thus, it is crucial to identify possible diagnosis tests to anticipate the progression of this disease.

Early diagnosis and treatment of disease progression in pregnant women, especially in the postpartum period, is important for preventing morbidity and mortality that may develop. Although tests related to diagnosis and progression are being investigated, these studies related to pregnancy are limited. In this study, we aimed to investigate whether there are possible laboratory tests associated with postpartum progression of COVID-19.

## Materials and methods

This retrospective study included patients with a positive COVID-19 test who gave birth in a tertiary medical centre between 01.03.2020 and 31.03.2021. The study protocol was approved by the local ethical review board of University of Health Science, Gazi Yaşargil Education and Research Hospital. Those who gave birth in our hospital with a gestational period of 28 weeks or later were included in the study. Clinical and laboratory findings of the patients during the prenatal day and on the day of disease progression were evaluated. The clinical findings of the patients were evaluated with control values for a two-week period after postpartum discharge and by phone contact for those who did not come for a follow-up appointment.

Real-time polymerase chain reaction (RT-PCR) and thorax computed tomography (CT) were performed for the patients whose prenatal clinical symptoms and signs included fever, flu-like symptoms (headache, pain and burning sensation in the throat and eye, nasal congestion, tearing, muscle joint pain), respiratory symptoms (cough, shortness of breath, chest pain, sweating), gastrointestinal (GI) symptoms (nausea-vomiting, diarrhoea, abdominal pain not related to pregnancy) and loss of taste and smell. Paients with a positive COVID-19 RT-PCR test or a negative COVID-19 RT-PCR test twice with 24–48 hour intervals and diagnosed with COVID-19 with the typical findings of COVID-19 thorax CT were included in the study. Patients with suspected COVID-19 contact and positive test results were also included in the study. Pregnant women with comorbid diseases, such as diabetes, hypertension, arrhythmia, gestational diabetes and hypertension, gestational cholestasis, preeclampsia, atypical haemolysis, elevated liver enzymes, low platelet (HEELP) syndrome and chronic liver disease were excluded from the study. The body mass index (BMI) of the patients was also calculated. The patients' vital signs, oxygen saturation, white blood cell (WBC) count, D-dimer, kidney function markers, inflammatory parameters and thorax CT findings were recorded. The thorax CTs of the patients were evaluated by 2 experienced radiologists, and they were classified into 4 grades: early, progression, pike and resolution.^7^ According to the classification made by the World Health Organization (WHO) and the clinical findings and thorax CT results, mild and moderate patients were grouped as non-severe, while severe and critical patients were grouped in the same way. 8

Patients with a positive PCR test but no symptoms and signs associated with COVID-19 were included in the asymptomatic group. Postpartum patients with advanced disease and patients who did not progress were examined separately. The groups that developed and did not develop postpartum disease progression were compared in terms of clinical course and laboratory findings.

## Statistical analysis

Statistical analysis was performed using SPSS version 22 software (IBM, Armonk, NY, USA). Categorical data were expressed as numbers and percentages, and the Chi-square test was used for comparison. The Shapiro-Wilk test was used to analyse the normal distribution. The normal distribution test was applied for the continuous parametric data. For numerical data, the Student's t-test was used for group comparison. The paired samples t-test was used for prenatal and postnatal numerical data for each group. Test results P < .05 were considered statistically significant.

## Results

The data of 151 out of 169 COVID-19-positive patients meeting the criteria were examined. Thirty-one of the prenatal patients had flu-like symptoms and 69 had respiratory symptoms. Moreover, 29 patients had additional loss of taste and sensation, 23 had fever and 18 had GI symptoms and signs. Thorax CT was performed in 98 patients. Ten patients had no involvement, 30 were progressed, 54 were in the early stage of the disease and 4 were in the peak stage. Fifty-one (33.7%) of the prenatal patients were in the asymptomatic group, 68 (45.1%) were in the non-severe group and 32 (21.1%) were in the severe group. Disease progression developed in 35 mothers (7 in the asymptomatic group, 12 in the non-severe group and 16 in the severe group) within an average of 3 days post-delivery. Moreover, 6 patients had flu-like symptoms, 29 had respiratory distress and 6 had fever. Thorax CT was performed in 35 patients. Six were in the early stage, 11 were in the progressed stage and 18 were in the peak stage.

In the prenatal evaluation, progression was mostly observed in the severe group (p < .05) (Table 1). There was no significant difference between the groups in terms of prenatal tomography grading and gestational age (p > .05) (Table 1). There was no age difference between the groups (Table 1). The mean BMI was higher in the progressed group (p < .05) (Table 1).

**Table 1:** Clinical characteristics of the patients in the prenatal admission

	Non-progressed N=116	Progressed N=35	P
Age (years)	28,9 ± 5.9	30,5 ±6.2	0.193
BMI	31,8 ±2.8	36,4 ±4.1	<0.001
Gestational age (week)	36.7±2.1	36.4±2.5	0.552
**Clinical stage**			
Asymptomatic	44 (37.9 %)	7 (20 %)	
Mild	32 (27.6 %)	6 (17.1 %)	
Severe	40 (34.5 %)	22 (62.9 %)	
**CT stage**			
Early	14 (26.6%)	4 (14.3 %)	
Progressed	37 (59.7 %)	21(75 %)	
Peak	3 (4.8 %)	1 (3.6 %)	
No involvement	8 (12.9 %)	2 (7.1 %)	

BMI; body mass index.

The WBC, lymphocyte and albumin levels increased, while the neutrophil lymphocyte ratio (NLR) and the neutrophil, LDH, CK, D-dimer, ferritin, CRP and IL-6 levels decreased in the non-progressed goup in comparison to the progressed group (p < .05) (Table 2). In the prenatal comparison of the progressed and non-progressed patients, the mean albumin was lower in the progressed group (p < .05) while the other laboratory findings were similar in both groups (p > .05) (Table 2). After delivery, the WBC, lymphocyte and albumin levels were lower in the progressed group, and the NLR and the neutrophil, LDH, CK, D-dimer, ferritin, CRP and IL-6 levels were higher in comparison to the prenatal findings (p < .05) (Table 2). In comparison to the prenatal period, the decrease in the WBC, lymphocyte and albumin levels and the increase in the NLR, and the neutrophil LDH, CK, CRP, IL-6, ferritin and D-dimer levels after delivery, were found to be predictive parameters with poor clinical course in showing postnatal progression (p < .05) (Table 2).

**Table 2:** Comparison of the prenatal and postpartum laboratory findings in the COVID-19 positive patients

	Prenatal		Postpartum			
			
	Non- progressed	Progressed	Non- progressed	Progressed	P Value**	P Value***
WBC	8.4±3.4	8.1±3.9	11.1±3	5.6±2.5	**<0.001**	**<0.001**
**P Value** *	0.673		**<0.001**			
LYM	13.4±6.5	11.6±5.9	15 ± 5.8	7.2±3.9	**<0.001**	**<0.001**
**P Value** *	0.153		**<0.001**			
NEU	82.7 ± 6.4	81.2±10.2	78.6±10.3	88.8±4.7	**<0.001**	**<0.001**
**P Value** *	0.297		**<0.001**			
NLR	8.5 ± 5.7	9.6 ± 5.9	6.3± 3.2	17.8± 11.8	**<0.001**	**<0.001**
**P Value** *	0.359		**<0.001**			
LDH	489±315	530±427	341±305	906±390	0.002	**<0.001**
**P Value** *	0.610		**<0.001**			
CK	109±115	160±184	106±119	320±224	0.727	**0.005**
**P Value** *	0.134		**<0.001**			
ALB	34.1±7.1	29.7± 3	37.3± 8	21.2± 4.3	**0.001**	**<0.001**
**P Value** *	**<0.001**		**<0.001**			
D-DIMER	709±373	941±1345	463±392	2529±1951	**<0.001**	**<0.001**
**P Value** *	0.320		**<0.001**			
FERRITINE	339±229	404±157	255±197	936±257	**0.009**	**<0.001**
**P Value** *	0.061		**<0.001**			
CRP	50.5±49.7	60.1±42.8	41.1±47.5	207.8±55	**0.007**	**<0.001**
**P Value** *	0.304		**<0.001**			
IL-6	25.4± 26.6	26.9±25.6	17.2± 16.9	101.6± 24.1	**0.003**	**<0.001**
**P Value** *	0.765		**<0.001**			

* Comparison of the non-progressed and progressed patients' findings in prenatal and postpartum term seperately

** Prenatal and postpartum comparison of the non-progressed patients' findings

*** Prenatal and postpartum comparison of the progressed patients' findings

Before delivery (prenatal period), COVID-19 progressed in 7 pregnant women in the asymptomatic group, 6 women in the non-severe group and 1 woman in the severe group. After delivery, the disease progressed in 12 women in the non-severe group, 10 in the severe group, and 2 in the critical group. Moreover, 16 women in the progressed group were in the critical group after delivery. The Caesarean section rate was 60.2% (91/151); 39.8% (60/151) of the deliveries were vaginal.

Eighteen patients from the progressed group were followed up in the intensive care unit for an average of 6 days. Two of the patients were given high flow oxygen (HFO), and 16 were given mechanical ventilation support, 5 of which were invasive. Two mothers died on the 16^th^ day and 29^th^ day of the follow-up due to multiorgan failure; both women were older (37 years and 38 years) and obese (BMI ≥ 30). Both these women underwent a Caesarean section in the preterm period (35 weeks and 36 weeks of gestation) and received invasive mechanical ventilation after undergoing the Caesarean section. Haemodialysis was applied due to kidney failure that developed in both of the women during follow-up. That was accompanied by toxic hepatitis and pulmonary embolism. In addition, 2 mothers in the severe group had sudden intrauterine infant death. Due to the presence of malposition, a Caesarean delivery was performed.

## Discussion

In SARS CoV-2-positive pregnant women that are asymptomatic or symptomatic, disease progression, ranging from aggregation of symptoms to death after giving birth, has been detected.^4^–^6^,^9^ To the best of our knowledge, our study was the first to reveal the predictive haematological and immunological parameters in pregnant women with COVID-19-positive disease progression. In this study, the most important parameters in showing poor clinical course and postpartum disease progression were decreased WBC, lymphocyte and albumin levels and increased neutrophil levels and NLR in comparison to the prenatal period.

Prabhu et al. observed clinical deterioration in 9 pregnant women within 7 days after delivery in their prospective study of 70 patients.^9^ Hantoushzadeh et al. stated that the group of 9 pregnant women with prenatal care progressed to the critical stage after giving birth, and 7 of them died while being followed up with mechanical ventilation in the postpartum period.^5^ In their analysis of 153 pregnant women with COVID-19-positive asymptomatic or mild symptoms in the prenatal period, An et al. found that hypoxia developed in 13 (7.8%) of the patients after giving birth and 3 of them after follow-up with advanced oxygen due to severe clinical manifestation after Caesarean section.^4^ Lymphopenia and elevated CRP were detected in the women in the early postpartum period who had respiratory distress and deoxygenation.^4^ In a national surveillance analysis, 124 of 978 acute respiratory distress syndrome (ARDS)-related maternal deaths had COVID-19, and 50 out of 143 occurred in the postpartum period. 6 In that report, half of the patients had no risk factors and the mean age of the non-survivors was not much higher than that of the survivors; thus, being healthy and young was not a protective factor for COVID-19-related death after giving birth. As we observed in our study and in different studies in the literature, the clinical situation in COVID-19-positive pregnant women may progress in the postpartum period and become worse.

Physiologically, it is unclear why these women developed serious symptoms after delivery. Postpartum hemodynamic, immunologic and plasma volume changes may induce pulmonary vascular permeability and, potentially, decompensation. Moreover, reduced immune system response, as seen in COVİD-19, may increase the inflammatory changes. Three days after delivery is a critical time because of the unstable periods of perinatal haemodynamic. An approximately 500 ml blood volume transfusion is required to improve pulmonary circulation and increase blood flow. Discharge of catecholamines and release of inflammatory mediators induce the fluid transport between the cellular compartments; this process may result in pulmonary oedema.^10^ Decreased albumin levels during the pregnancy and delivery process lead to a decrease in colloidal osmotic pressure that induces fluid leakage from the vessels.^11^ Increased fluids in the pulmonary alveoli can reduce the oxygen exchange and oxygenated blood flow. Viral pneumonia, as seen in COVID-19, may exaggerate these changes and induce the occurrence of ARDS.^12^ Delivery also promotes cytokine secretion, and it may increase the severity of COVID-19.^13^ These all changes during the short period of the postpartum process can affect the survival of a mother who has COVID-19. Laboratory tests can become more important during and before this critical period. In our study, we observed statistically higher BMI and lower albumin levels in the progressed group at the first diagnosis. In the progressed group, the albumin levels after delivery were dramatically decreased. As seen in the information presented above, lower albumin levels may be a warning sign of the need for intense follow-up after delivery in a patient with COVID-19.

Yang et al. compared the prenatal and postnatal laboratory findings of 13 COVID-19-positive patients in the asymptomatic or mild group and 42 COVID-19-negative patients without disease progression in the postpartum period.^14^ During the prenatal and postpartum periods, there was no difference in the WBC, neutrophils and lymphocyte levels, the NLR and the level of CRP between the confirmed COVID-19 group and the control group.^14^ In a study involving a total of 548 COVID-19 patients, including 345 mild/moderate, 155 severe and 48 critical cases, increased NLR and increased levels of CRP, IL-6, ferritin and D-dimer with lymphopenia were found to be associated with reduced survival.^15^ Severe outcomes, such as ARDS and death, are associated with massive neutrophil infiltration in the lung and dramatically elevated neutrophil counts in the peripheral blood.^16^ A significant decrease in lymphocyte count has been mentioned to be related to the disease severity of SARS and COVID-19.^17^,^18^ The NLR has been reported to be an independent predictor of disease severity in patients with COVID-19.^15^,^19^ We confirmed the observation of higher initial NLR in severe/critical patients, and we further evaluated the potential of NLR as a prognostic factor. In our study, there was no difference between the progressed and the non-progressed groups regarding NLR in pregnancy, but NLR was significantly increased in the progressed group after delivery. The increase in the NLR after delivery may be an indication of postpartum COVID-19 progression.

Increased proinflammatory cytokine/chemokine responses, including IL-6, has been implicated in human coronavirus pathogenesis.^20^ High levels of these cytokines/chemokines are considered to lead to tissue damage, accounting for respiratory failure or multiorgan failure. In several reports, 21,22 higher levels of IL-6 were found in the non-survivors, and this data may show the association between the severity of the COVID-19 and IL-6 levels. In another study that investigated COVID-19, ferritin was also found to be higher in the non-survivors on admission in comparison to the survivors.^15^ In our study, we observed that the inflammatory markers, such as ferritin, IL-6, CRP and D-dimer levels, were similar in the progressed and non-progressed COVID-19 groups on admission for pregnancy. While these markers improved rapidly after giving birth in the non-progressed group, they continued to increase in the progressed group. We think that the follow-up of these parameters during the follow-up period may be useful in predicting the prognosis of this disease.

Our study has some limitations. First, the study design was retrospective and the sample size was small. Second, we only investigated the serum IL-6 levels from the T-cell response indicators.

## Conclusion

We observed that prenatal low albumin and high BMI may be related to the progression of COVID-19 in pregnant women after delivery. In the progressed group, the inflammatory markers were increased after delivery; in the non-progressed group, they were improved. These markers may be an indication of the postpartum progression of COVID-19. More comprehensive studies are needed to obtain more information about this subject.

## Source(s) of support

Nil.
